# Project-based learning with arduino robots: impact on undergraduate students’ achievement and task persistence in robotics programming

**DOI:** 10.3389/frobt.2025.1615427

**Published:** 2025-07-10

**Authors:** Fadip Audu Nannim, Nnenna E. Ibezim, Moeketsi Mosia, Basil C. E. Oguguo

**Affiliations:** ^1^ Department of Mathematics, Natural Sciences and Technology Education, University of the Free State, Bloemfontein, South Africa; ^2^ Department of Computer and Robotics Education, University of Nigeria, Nsukka, Nigeria; ^3^ Department of Science Education, University of Nigeria, Nsukka, Nigeria

**Keywords:** project-based, arduino, students achievement, task persistence, robotics programming

## Abstract

**Introduction:**

Programming is a fundamental skill in the 21st century, yet there is a global shortage of skilled programmers for high-tech jobs. This study determined the effects of Project-Based Arduino Robot Application (PARA) on undergraduate students’ achievement and task persistence in robotics programming.

**Methods:**

The quasi-experimental research design was adopted for the study. A sample of 74 second-year computer and robotics education students from three intact classes in three tertiary institutions offering robotics programming II were selected forthe study.

**Results and Discussion:**

PARA improved the academic achievement of students in robotics programming (63.00 ± 16.81) more than the conventional method, which uses Interactive PowerPoint (IPP) (43.79 ± 12.07). PARA improved the task persistence of students in robotics programming (73.75 ± 13.46) more than the conventional method (40.00 ± 13.70). Male students taught robotics programming using PARA had a slightly higher mean achievement score (69.60 ± 11.50) than their female counterparts (52.00 ± 19.43). Female students taught robotics programming using PARA had a slightly higher mean task persistence score (78.67 ± 11.96) than their male counterparts (70.80 ± 14.02). There was a significant difference (p < 0.05) in students’ mean achievement scores based on the instruction method used in teaching robotics programming, among others. These findings have implications for instructing students who find robotics programming difficult and abstract.

## 1 Introduction

The rapid advancement of robotics and artificial intelligence (AI) has revolutionized various sectors, including education. As industries increasingly demand graduates with computational thinking and programming proficiency, universities are under pressure to equip students with relevant skills in robotics programming (Belmar, 2022). However, despite the growing importance of robotics education, many students struggle with programming concepts due to their abstract nature, leading to low achievement and persistence in robotics-related courses (Evripidou et al., 2020). This challenge necessitates a shift from traditional lecture-based instruction to more interactive, hands-on approaches that can enhance students’ engagement, comprehension, and problem-solving skills. One promising instructional approach that has gained attention in recent years is project-based learning, particularly through the integration of Arduino robotics applications.

Existing research has explored various instructional strategies for teaching robotics programming. Studies have demonstrated that project-based learning enhances students’ problem-solving abilities, critical thinking, and overall engagement (Nannim et al., 2024; Marouani, 2022; Papadakis, 2020). Additionally, Papert’s constructionist theory posits that learners construct knowledge more effectively when actively engaged in hands-on experiences (Papert, 1993). While these studies have established the benefits of active learning in robotics education, gaps remain in understanding how gender differences influence achievement and persistence in programming when project-based approaches are employed. Some studies suggest no significant gender differences in programming achievement (Zhong et al., 2016; Akinola, 2015), while others report conflicting results (Noh and Lee, 2020; Ayalew et al., 2018). Moreover, previous research has not sufficiently examined how project-based Arduino applications specifically impact both achievement and task persistence in robotics programming, nor how instructional strategies can mitigate gender disparities in this field.

**FIGURE 1:**
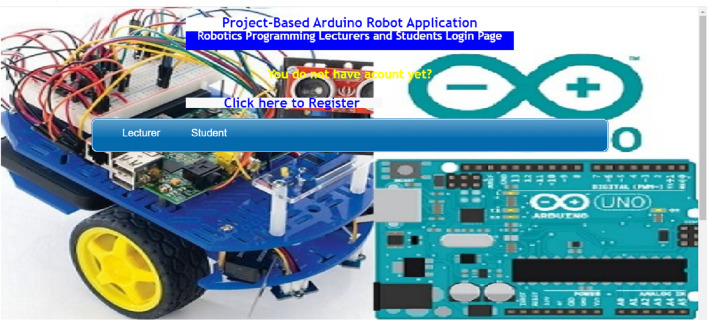
Home page (source: Nannim et al., 2025).

**FIGURE 2:**
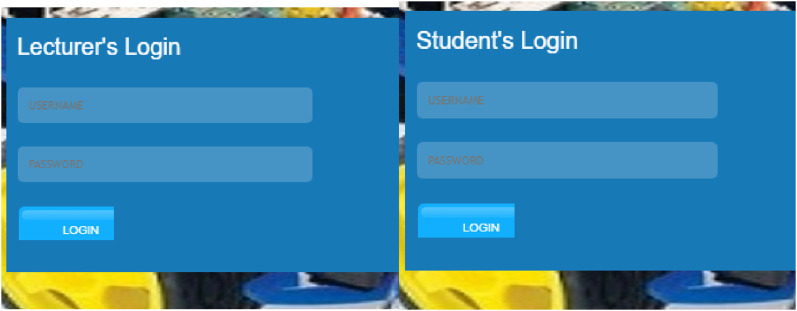
Lecturers’ and students’ login (source: Nannim et al., 2025).

**FIGURE 3:**
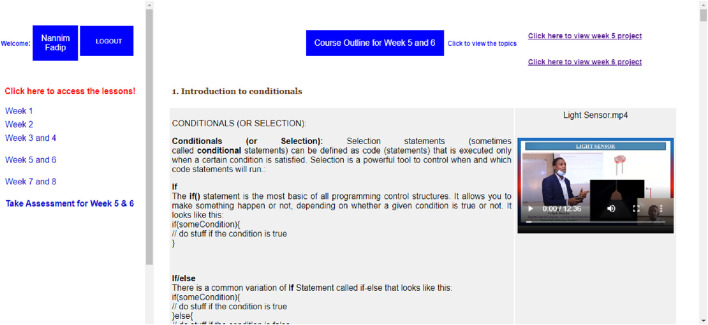
Course outline/content page (source: Nannim et al., 2025).

To address these gaps, this study investigates the effect of the Project-Based Arduino Robot Application (PARA) (Nannim et al., 2025) on undergraduate students’ achievement and task persistence in robotics programming. The Project-Based Arduino Robot Application (PARA) is a novel pedagogical method aimed at learning robotics programming via hands-on, student-centred activities rooted in Papert’s constructionism (Nannim et al., 2025). PARA integrates practical problem-solving with concrete computing by engaging students in the construction and programming of Arduino-based robotic devices. It is a structured digital educational platform that includes login interfaces for both instructors and students. Instructors may post information including videos, graphics, quizzes, and weekly projects, while students sequentially access instructional resources by completing quizzes and practical projects (see Figures 1–3).

The content in PARA advances from fundamental concepts, encompassing microcontroller fundamentals, circuit design, and C/C++ syntax, to advanced projects involving sensor integration, looping constructs, conditional logic, and wireless control utilising components like photocells, PIR sensors, temperature sensors, IR remote systems, and Bluetooth modules. Each course includes instructional videos that support learners by incorporating a multimedia component, in line with Mayer’s Cognitive Theory of Multimedia Learning, which posits that students learn more effectively when they can hear, see, manipulate, and actively engage with educational content rather than relying on a single mode of instruction, such as listening or viewing alone (Mayer, 2005).

The Project-Based Arduino Robot Application (PARA) distinguishes itself through a structured, curriculum-aligned, and technology-enhanced instructional model. In contrast to conventional lecture-based methods or simulation-only robotics programs, PARA integrates continuous access to physical computing components, multimedia scaffolding, and embedded formative assessments within a modular digital platform. This combination reduces the cognitive load often associated with abstract programming concepts while aligning closely with curricular goals. Unlike many project-based learning (PBL) models that lack standardisation or sustained access, PARA offers a uniquely inclusive and consistent learning pathway that not only fosters computational thinking and task persistence but also ensures measurable academic outcomes. This synthesis of constructionist learning, multimedia design (Mayer, 2005), and structured progression represents a meaningful innovation in robotics programming pedagogy. By comparing PARA with conventional lecture-based instruction, this research aims to determine its effectiveness in enhancing learning outcomes and fostering long-term engagement in robotics education. Furthermore, this study examines whether PARA influences gender disparities in achievement and persistence, contributing to a more inclusive approach to robotics education. Findings from this research will provide valuable insights for educators, curriculum developers, and policymakers in designing more effective instructional strategies for teaching robotics programming at the university level.

### 1.1 Literature review

Programming is the process of designing and developing executable computer instructions to address specific computational needs or perform designated tasks. It is also defined as the practice of instructing electronic machines to execute tasks, solve problems, and enable human-computer interaction (Bebbington, 2014). Additionally, programming involves writing lines of code that guide computers, applications, or robots in their operation. A robot, as defined by Ben-Ari and Mondada (2018), is an intelligent and adaptable machine designed to perform tasks autonomously by executing programmed instructions, often with the aid of sensors that enable it to perceive and respond to its environment. The process of coding instructions to control the behaviour of a robot or any complex automated system is known as robot programming (MathsWorks, 2022; Joseph, 2020), often referred to as robotics programming. Robotics, in a broader sense, encompasses the design, development, implementation, and operation of robots. It is an interdisciplinary field integrating artificial intelligence, automation, sensor technology, mechanical and electrical engineering, and computer science (Cai, 2011).

Robotics programming, hereafter referred to as “programming” in this study, includes tasks such as algorithm analysis, algorithm generation, accuracy profiling, resource optimization, and execution, commonly known as coding (Mohamed, 2020). Additional activities involve testing, debugging, maintaining source code, implementing designed systems, and managing a computer’s machine code (Agarwal et al., 2010). Acquiring programming skills opens pathways to high-paying careers globally (ComputerScience, 2021; Mulas et al., 2017). However, research indicates that learning to program is highly challenging (Ishihara and Rattanachinalai, 2021; Gurer and Tokumaci, 2020; Nurul et al., 2020; Massoudi, 2019). Programming requires an understanding of syntax and semantics, variable manipulation, data types, arithmetic and relational operations, and control structures. Mastering these elements is difficult for many students, making programming a cognitively demanding and time-intensive endeavour (Tupac-Yupanqui et al., 2022; Ubaidullah et al., 2021; Ishihara and Rattanachinalai, 2021).

The difficulty associated with programming is reflected in student dropout rates in computer science-related disciplines. According to the Higher Education Statistics Agency (HESA, 2022), computer science recorded the highest dropout rate (9.8%) among all academic disciplines in the 2021/2022 academic year, followed by Business and Administrative Studies (7.4%) and Engineering and Technology (7.2%). Margulieux et al. (2020) reported that failure and dropout rates in introductory programming courses could reach as high as 50%. The primary reason for these high dropout rates is the complexity of learning programming, which is central to computing education (Safta and Stan, 2020; Kwon et al., 2020).

Similarly, studies conducted in Nigerian tertiary institutions have revealed high failure rates in programming courses. Asogwa (2017) reported that out of 354 students enrolled in programming courses between 2013 and 2016, the following grades were recorded: A (0%), B (9%), C (24%), D (10%), E (14%), and F (43%). A related study by Olelewe (2015) in two tertiary institutions in Enugu State found that only 821 (55.92%) out of 1,468 students successfully passed programming courses over 6 years. More recent data from the 2019/2020 academic session at the Department of Computer and Robotics Education, University of Nigeria, Nsukka, revealed that no student obtained an “A” grade in a programming course. The distribution of grades was as follows: B (7.69%), C (42.31%), D (23.08%), E (7.69%), F (7.69%), while 11.54% of students had incomplete results due to failure to submit continuous assessments or abandonment of the course.

The increasing dropout rate in computing disciplines is primarily attributed to the high failure rates in introductory programming courses (Dasuki and Quaye, 2016). Contributing factors identified by Dasuki and Quaye (2016) include a lack of intrinsic motivation, programming anxiety, and ineffective teaching strategies. Adewale (2020) also found that persistent failure in programming courses among Nigerian undergraduates is linked to poor pedagogical approaches. Despite the conventional use of Interactive PowerPoint (IPP) as a teaching method for robotics programming, the challenge of effective learning remains prevalent.

The conventional lecture method using an Interactive PowerPoint (IPP) in robotics programming necessitates the use of a specially designed instructional package. According to Taub (2019), an interactive training package consists of adaptable training materials that enhance learning and comprehension of abstract concepts, particularly in science, technology, and computing. The IPP serves as a teaching aid for both lecturers and students in robotics programming, facilitating engagement through interactive elements such as options, guidelines, menus, and various user interface (UI) controls. However, despite its application, there is no substantial evidence of improved student achievement in robotics programming among undergraduates in Southeast Nigeria.

A major challenge in programming education is the need to shift from a theoretical approach to a more engaging and interactive learning experience (Perenc et al., 2019). This challenge is exacerbated by the shortage of adequately trained instructors with the necessary pedagogical and content knowledge to teach programming effectively (Scherer et al., 2020). Research also indicates that many lecturers employ monotonous, non-engaging, and non-collaborative teaching methods, which fail to capture students’ interest (Kagombe et al., 2019; Massoudi, 2019). Given that 21st-century students, often referred to as digital natives, are accustomed to technologies such as computers, tablets, and gaming consoles (Gurung and Rutledge, 2014; Koumachi, 2019), traditional programming instruction, such as the introductory “Hello World” approach, no longer appeals to them (Gurung and Rutledge, 2014). Instead, students prefer engaging, thought-provoking content that is relevant to real-world applications (Sparf et al., 2022). This underscores the need for pedagogical innovations like project-based learning (PBL) to make programming education more interactive and student-centred.

PBL is a dynamic instructional approach in which students actively explore real-world problems, fostering deeper learning and skill acquisition. Krajcik and Blumenfeld (2006) state that PBL is grounded in constructivist theory, emphasizing active knowledge construction through inquiry, problem-solving, and collaboration. Through this approach, students investigate questions, propose hypotheses, engage in discussions, and experiment with new projects, ultimately strengthening their programming competence. Empirical studies have shown that PBL enhances students’ academic achievement in science and mathematics more effectively than traditional instruction (Chen and Yang, 2019; Condliffe, 2017; Guo et al., 2020). However, limited research exists on the impact of PBL on student achievement in programming education (Chen and Yang, 2019). PBL has also been found to boost student engagement, motivation, and self-efficacy (Chis et al., 2018; Al Said et al., 2019). Despite its benefits, the evaluation of PBL effectiveness is often constrained by a lack of valid and reliable assessment measures for the deep learning and competencies it promotes (Condliffe, 2017).

A promising PBL strategy for enhancing students’ programming engagement, task persistence, computational ability, and overall achievement is the integration of Arduino robotics (Ntourou et al., 2021; Yilmaz-Ince and Koc, 2021). Several educational robotics kits such as Lego Mindstorms Education EV3, Lego Spike Education, Raspberry Pi, and Arduino have been widely used in different educational levels to facilitate programming instruction (Piedade et al., 2020; Skerl, 2020; Flanagan, 2020; Rossano et al., 2020). Among these, Arduino stands out as an open-source electronics platform that offers affordability, flexibility, and compatibility with a wide range of electronic components (Arduino, 2021). The Arduino board processes inputs and generates outputs through programmed code, making it highly adaptable for diverse projects. With extensive open-source libraries, Arduino supports applications ranging from simple to highly complex projects, including robotics, remote-controlled vehicles, and smart home devices (Domazetovska et al., 2019; Fidai et al., 2020). Due to its simplicity and affordability, Arduino is particularly well-suited for beginner programmers. Rossano et al. (2020) emphasize that real-object programming is more effective, as hands-on interaction with physical components enhances motivation and fosters algorithmic thinking beyond mere code generation.

The educational benefits of Arduino robotics have been extensively documented, and studies have highlighted its effectiveness in teaching computer related skills (Marín-Marín et al., 2024; Nannim et al., 2025) and its impact on learning science and mathematics (Karaahmetoğlu and Korkmaz, 2019; Morón et al., 2019). Some of these studies suggest that Arduino robotics can make programming more engaging and enjoyable for students struggling with abstract concepts (Papadakis, 2020; Kagombe et al., 2019; Massoudi, 2019). A tailored PBL approach incorporating Arduino robotics can transform programming education into an interactive and stimulating learning experience (Karaahmetoğlu and Korkmaz, 2019; Rahul et al., 2014).

Arduino-based robotics projects utilize components such as Light Emitting Diodes (LEDs), potentiometers, temperature sensors (thermistors), Passive Infrared (PIR) sensors, servo motors, water level sensors, buzzers, humidity sensors, Liquid Crystal Displays (LCDs), and light sensors (photocells), among others. Hands-on projects such as LED flashing, traffic light control, LED dimming, RGB diode programming, and photoresistor-controlled LED activation can significantly improve students’ programming skills by providing tangible, real-world applications (Chettaoui et al., 2022; Burbaite et al., 2013). This active learning approach ensures that students remain engaged and develop a deeper understanding of programming concepts.

The theoretical foundation for integrating Arduino into PBL is grounded in Papert’s constructionism theory, which extends constructivism by emphasizing learning through tangible, real-world applications (Papert, 1980). Constructionism posits that students acquire knowledge most effectively through hands-on problem-solving and project-based activities. Robotics projects with Arduino provide an ideal environment for students to construct solutions to programming challenges while simultaneously developing essential computational skills. This approach can potentially enhance students’ academic achievement in programming-related courses by fostering persistence, engagement, and critical thinking.

Academic achievement is broadly defined as students’ attainment of educational goals, typically measured through assessments, examinations, and continuous evaluations (Ward et al., 1996). Spinath (2012) describes academic achievement as performance outcomes in intellectual domains across different educational levels. As a key indicator of educational quality, academic achievement is crucial for individual and societal progress. However, research consistently highlights low achievement levels in programming courses (Ishihara and Rattanachinalai, 2021; Gurer and Tokumaci, 2020; Kagombe et al., 2019). Addressing these challenges requires adopting innovative teaching methods that enhance student engagement and learning outcomes. A PBL approach incorporating Arduino robotics holds significant promise in mitigating learning difficulties, sustaining student interest, and improving academic achievement in programming education.

Task persistence, also known as perseverance, refers to the ability to remain engaged in an activity despite physical or emotional discomfort, distractions, or a lack of immediate success. DiCerbo (2016) defined task persistence as continuing an activity in the face of difficulties, obstacles, or failure. Dumdumaya and Rodrigo (2018) found that students’ engagement, measured by their average time on task and self-efficacy, represented by the average number of reattempts, time spent on resources after failure, and the proportion of difficult problems attempted, are key factors influencing persistence. Similarly, Israel-Fishelson and Hershkovitz (2019) described task persistence as a learner’s determination to complete a learning process and achieve their educational goals. According to Allsopp and Ejsing-Duun (2016), learning to program is a cognitively demanding task that requires perseverance due to its reliance on complex abstractions and mathematical reasoning. Consequently, an active educational context that fosters motivation and engagement is essential to sustain students’ persistence in learning programming. This active learning approach will likely enhance motivation, enjoyment, commitment, and enthusiasm while improving computational practices (Papadakis, 2020).

However, previous studies have suggested that students’ academic achievement in programming may be influenced by gender (Noh and Lee, 2020; Malik and Coldwell-Neilson, 2018; Ayalew et al., 2018; Baser, 2013; Gunbatar, 2018). Gender, from a general perspective, encompasses the socially constructed roles and behaviours typically associated with males and females. The World Health Organization (2021) defines gender as a sociocultural constructed characteristic and role assigned to individuals based on their perceived societal identity. Research on the influence of gender on computer programming performance has yielded inconclusive results (Noh and Lee, 2020; Malik and Coldwell-Neilson, 2018; Gunbatar, 2018). Some studies have reported significant differences in programming performance between male and female students. For instance, Ayalew et al. (2018) and Baser (2013) found that male students outperformed their female counterparts in computer programming. Conversely, Noh and Lee (2020) reported that female students achieved higher performance levels than their male counterparts. Other studies have indicated no significant gender differences in programming performance (Zhong et al., 2016; Akinola, 2015; Lau and Yuen, 2009). Sharma and Shen (2018), in a comparative study of Indian and Australian universities, found that while there was no gender-based performance difference among Australian students, male students in Indian universities significantly outperformed their female counterparts. These mixed findings suggest that the influence of gender on academic achievement in programming remains inconclusive.

Given the importance of task persistence and academic achievement in programming, alongside the potential moderating role of gender, it is crucial to examine how these factors interact in the context of robotics programming. This study is a part of a PhD dissertation which investigates the effects of a Project-Based Arduino Robot Application (PARA) on students’ achievement, computational skills, and task persistence in robotics programming courses in tertiary institutions in Southeast Nigeria.

### 1.2 Purpose of the study

The primary purpose of this study is to determine the effects of PARA on undergraduate students’ achievement and task persistence in robotics programming.

### 1.3 Hypotheses

The following hypotheses were formulated and tested at a 5% significant level.

Ho_1_: There is no significant difference in the mean achievement scores of students taught robotics programming with PARA and those taught with IPP.

Ho_2_: There is no significant difference in the mean ratings of task persistence of the students taught robotics programming with PARA and those taught with IPP.

Ho_3_: Gender does not significantly influence the mean achievement scores of students in robotics programming when taught using PARA.

Ho_4_: Gender does not significantly influence the mean ratings on task persistence of students taught robotics programming using PARA.

Ho_5_: There is no significant interaction effect of teaching methods and gender on the mean achievement scores of students in robotics programming.

Ho_6_: There is no significant interaction effect of teaching methods and gender on students’ mean ratings on task persistence in robotics programming.

## 2 Methods

### 2.1 Design of the study

The study employed a pretest-posttest non-equivalent control group quasi-experimental research design. In quasi-experimental research, intact groups are used when it is not feasible to randomly assign participants due to institutional or logistical constraints (Creswell, 2012). As a result, quasi-experimental designs do not offer full control over extraneous variables, as they lack random assignment of subjects to groups. However, this design was deemed appropriate for the study since intact classes were utilised to prevent disruptions to the regular instructional schedule.

### 2.2 Participants

The study sample comprised 74 second-year Computer and Robotics Education students from three intact classes across three tertiary institutions in Southeast Nigeria that offer Robotics Programming II. The selected institutions were the University of Nigeria, Nsukka (Enugu State), Alvan Ikoku Federal University of Education (AIFUE), Owerri (Imo State), and Nwafor Orizu College of Education, Nsugbe (Anambra State). These institutions were selected using a simple random sampling technique. Additionally, the treatment and control groups were assigned through simple random sampling. As a result, the University of Nigeria, Nsukka, and Nwafor Orizu College of Education, Nsugbe, formed the treatment group, while AIFUE Owerri, served as the control group. Nwafor Orizu College of Education, Nsugbe, is an affiliated college of the University of Nigeria, Nsukka. The selected institutions offer the same degree program and follow the same curriculum. Therefore, the three institutions were considered comparable and suitable for the study.

The treatment group consisted of 35 students (20 males and 15 females), while the control group comprised 39 students (24 males and 15 females), bringing the total sample size to 74. This sample size was deemed adequate for the experimental study. According to Creswell (2012), an educational experiment typically requires a minimum of 15 participants per group as a rough guideline for an appropriate sample size.

### 2.3 Instrument

Two instruments were used for data collection: the Robotics Programming Achievement Test (RAT) and the Task Persistence Scale (TPS). The researcher developed the RAT based on the Robotics Programming II syllabus (CRE 252), a 200-level course. It comprised 25 multiple-choice items, each carrying four marks. The test was administered as both a pre-test and post-test to assess students’ achievement in robotics programming. The allotted time for the test was 20 min. A marking scheme was prepared and used for scoring, while a test blueprint (table of specifications) guided the construction of the test items. The blueprint was based on the revised Bloom’s cognitive taxonomy, with 60% (14 items) categorised as easy (Remembering and Understanding), 30% (8 items) of moderate difficulty (Applying and Analysing), and 10% (3 items) classified as difficult (Evaluating and Creating). The questions and answer options were reshuffled during the post-test administration to minimise familiarity with the test items.

The Task Persistence Scale (TPS) was a self-developed instrument designed by the researcher through a review of relevant literature. It was structured as a 7-point Likert scale, with response options ranging from 1 = Strongly Disagree to 7 = Strongly Agree. Items 1 to 4 assessed students’ reactions to robotics programming tasks, while Items 5 to 14 measured their persistence in completing robotics programming-related tasks. The TPS was used to evaluate students’ level of task persistence in robotics programming.

### 2.4 Validation of the instrument

The instruments (Robotics Programming Achievement Test (RAT) and Task Persistence Scale (TPS)), along with the study’s purpose, research questions, and hypotheses, were subjected to face validation by five experts three from the Department of Computer and Robotics Education and two from the Educational Measurement and Evaluation Unit, Science Education Department, all at the University of Nigeria, Nsukka. The validators assessed the instruments based on their alignment with the study’s purpose, grammatical correctness, clarity and ambiguity of items, and the suitability of the research questions and hypotheses.

The RAT underwent content validation using a test blueprint (Table of Specifications) to ensure adequate coverage of the course syllabus. The TPS was subjected to construct validation to determine the appropriateness of its items. To achieve this, 50 copies of the TPS were administered to 50 second-year students in the Department of Computer Science, University of Nigeria, Nsukka. After the administration and retrieval of responses, factor analysis was conducted using the Principal Component Analysis (PCA) in SPSS version 23.

Three factors were extracted for the TPS based on the Scree Plot generated by SPSS, and the Varimax Rotation technique was applied. To determine the validity of the items, the Rotated Component Matrix was examined using Meredith’s (1969) benchmark of 0.35 and above for valid factor loadings. According to Meredith, only factor loadings of 0.35 and above on a single factor should be considered valid. Based on this criterion, out of the 20 initial items in the TPS, 14 items (Items 1, 2, 5, 7, 9, 10, 12, 13, 15, 16, 17, 18, 19, and 20) loaded exclusively on Factor I and were considered factorially pure.

No factorially pure items were found for Factors II and III. Items 3, 4, 6, and 14 had loadings of 0.35 and above on two or all three factors, making them factorially complex; they were therefore removed. Additionally, Items 8 and 11 were considered factorially impure, as they had loadings below 0.35 on all factors, and were also discarded. Consequently, the final version of the TPS comprised 14 validated items, which were renumbered sequentially.

### 2.5 Reliability of the instrument

The reliability of the instruments was determined through a trial test conducted on 15 second-year undergraduate students from the Department of Computer and Robotics Education, Federal College of Education Omoku, River State, who were not part of the main study. The selection of second-year students was appropriate because they take similar robotics programming courses. For the Robotics Programming Achievement Test (RAT), the internal consistency reliability index was calculated using the Kuder-Richardson Formula 20 (KR-20), yielding a reliability coefficient of 0.80. Similarly, for the Task Persistence Scale (TPS), the internal consistency reliability coefficient was determined using Cronbach’s Alpha, resulting in a value of 0.70. These reliability indices indicate that both instruments are highly reliable and effectively measure the intended constructs.

### 2.6 Experimental procedure

The pre-achievement test and task persistence questionnaire were administered to both the control and experimental groups before the treatment to establish baseline data. The two groups underwent instruction for 8 weeks. The instructional content covered in the course included foundational topics such as an introduction to the Arduino microcontroller, resistor colour codes, circuit diagram interpretation, wiring of Arduino robots, and basic C/C++ syntax. Students engaged in Arduino robotics projects that illustrated sequential flow of control, including tasks such as programming an LED to flash, simulating traffic control using LEDs, generating tones with a buzzer or piezo speaker, reading analog input from a potentiometer (e.g., a light dimmer), and temperature sensing. Additionally, students explored conditional/selection statements (IF/ELSE IF/ELSE and SWITCH CASE) through hands-on projects using light sensors (photoresistors/photocells). Other key topics included a burglar alarm system using a Passive Infrared (PIR) sensor, controlling an LED with an infrared (IR) remote sensor, programming an Arduino robot to toggle a light bulb using an IR remote, and wirelessly controlling robots with smartphones via the HC-05 Bluetooth module. At the end of the teaching period, the post-achievement test and task persistence questionnaire were administered to both groups. The data collected from the pre- and post-achievement tests, as well as the task persistence questionnaire, were then analysed.

#### 2.6.1 Training of the lecturers

A 2-day training program was conducted for the computer and robotics education lecturers who assisted in the study, particularly those instructing the experimental group. The training focused on equipping them with the necessary skills to implement the Project-Based Arduino Robot Application (PARA) in guiding student learning. In contrast, lecturers in the control group followed a structured lesson plan.

#### 2.6.2 Control of extraneous variables

The researchers controlled the following extraneous variables: teacher-related variables, instructional setting variables, inter-group variables, and the effects of pre-test and post-test. To ensure that teacher quality did not influence the study’s findings, only computer and robotics education lecturers who regularly teach robotics programming were selected as research assistants. These lecturers were trained to implement the Project-Based Arduino Robot Application (PARA) and deliver instruction using the prepared lesson notes. The experimental and control groups followed a structured lesson package on the selected topics. The researcher conducted regular supervision to ensure adherence to the instructional plan.

To mitigate the influence of instructional setting variables, all lecturers utilised the researcher-prepared lesson notes as instructional guides throughout the study. Additionally, all students received instruction in their regular classrooms to maintain consistency in the learning environment. To account for the non-equivalence of intact classes, Analysis of Covariance (ANCOVA) was employed to adjust for initial differences between groups, addressing the lack of randomization. The interval between pre-test and post-test administration was set at 8 weeks to minimize the likelihood of students becoming familiar with the test items. To control the Hawthorne Effect, regular lecturers taught students in their usual classrooms during their scheduled robotics programming lessons. Furthermore, placing the treatment and control groups in separate tertiary institutions helped minimize potential biases related to students’ awareness of different instructional conditions.

## 3 Data analysis

The research questions were addressed using mean and standard deviation, while the hypotheses were tested using Analysis of Covariance (ANCOVA) at a 5% significance level (α = 0.05). ANCOVA was selected as the appropriate statistical tool because the study adopted a quasi-experimental design, specifically the non-equivalent control group design. This design incorporates pre-tests, which serve as covariates, allowing ANCOVA to adjust for initial differences and establish the equivalence of the groups before treatment. Additionally, since intact classes were used, ANCOVA enhances the power of the test by controlling for potential errors arising from the non-randomisation of participants.

For decision-making, mean gains were used to interpret the research questions. In hypothesis testing, an alpha (α) value of less than 0.05 (α < 0.05) indicates a statistically significant difference, leading to the rejection of the null hypothesis. Conversely, an alpha (α) value of 0.05 or greater (α ≥ 0.05) indicated no significant difference, resulting in the retention of the null hypothesis.

## 4 Results

The results presented in Table 1 indicate that students in the treatment group had a pre-test mean achievement score of (x̅ = 29.75, SD = 12.56) and a post-test mean achievement score of (x̅ = 63.00, SD = 16.81). In comparison, the control group recorded a pre-test mean achievement score of (x̅ = 30.77, SD = 9.82) and a post-test mean achievement score of (x̅ = 43.79, SD = 12.07). Furthermore, the adjusted mean achievement score for the treatment group was 61.78, whereas the control group had an adjusted mean score of 43.81. These findings suggest that students taught using the Project-Based Arduino Robot Application (PARA) outperformed those taught using the conventional lecture method (IPP), as reflected in their higher mean achievement scores.

**TABLE 1:** Achievement Score of students taught Robotics Programming Using Project-Based Arduino Robot Application (PARA) and those taught with Interactive PowerPoint (IPP).

Methods	N	Pre-test		Post-test		Adjusted mean ( $\left.\bar{x}\right)$
		$\bar{x}$	SD	$\bar{x}$	SD	
PARA	34	29.75	12.56	63.00	16.81	61.78
IPP	39	30.77	9.82	43.79	12.07	43.81

Table 2 shows a statistically significant main effect of the instructional method on students’ mean achievement scores in robotics programming, F (1, 68) = 47.098, p < 0.05. Consequently, the null hypothesis was rejected, indicating a significant difference in students’ mean achievement scores based on the instructional method used in teaching robotics programming.

**TABLE 2:** Analysis of covariance of students’ mean achievement scores in robotics programming.

Source	Type III Sum of Squares	df	Mean Square	F	Sig	Partial Eta Squared	Decision
Corrected Model	12894.340^a^	4	3223.585	28.212	0.000	0.624	Significant
Intercept	8520.469	1	8520.469	74.570	0.000	0.523	Significant
RATPreTest_Score	4019.126	1	4019.126	35.175	0.000	0.341	Significant
Method	5381.512	1	5381.512	47.098	0.000	0.409	Significant
Gender	696.756	1	696.756	6.098	0.066	0.066	Not Significant
Method * Gender	1201.927	1	1201.927	10.519	0.002	0.134	Significant
Error	7769.769	68	114.261				
Total	220560.000	73					
Corrected Total	20664.110	72					

a. R Squared = .624 (Adjusted R Squared = .602).

Table 3 shows that students in the treatment group (taught using PARA) had a pre-test mean task persistence score of (x̅ = 38.31, SD = 13.81) and a post-test mean task persistence score of (x̅ = 73.75, SD = 13.46). In contrast, students in the control group (taught using IPP) had a pre-test mean task persistence score of (x̅ = 43.08, SD = 19.04) and a post-test mean task persistence score of (x̅ = 40.00, SD = 13.70). Additionally, the adjusted mean scores for the treatment and control groups were 75.41 and 40.18, respectively. These findings suggest that students taught using PARA demonstrated significantly higher task persistence than those instructed using the conventional lecture method (IPP).

**TABLE 3:** Task Persistence Score of Students taught Robotics Programming using PARA and those taught with Conventional Lecture Method (IPP).

Methods	N	Pre-test		Post-test		Adjusted mean ( $\left.\bar{x}\right)$
		$\bar{x}$	SD	$\bar{x}$	SD	
PARA	34	38.31	13.81	73.75	13.46	75.41
IPP	39	43.08	19.04	40.00	13.70	40.18

The results in Table 4 show a statistically significant main effect of the instructional method on students’ mean task persistence scores in robotics programming, F (1, 68) = 155.662, p < 0.05. Consequently, the null hypothesis was rejected, suggesting a significant difference in task persistence between students taught using PARA and those taught using the conventional lecture method, with the PARA group demonstrating higher task persistence.

**TABLE 4:** Analysis of covariance of students’ mean ratings of task persistence in robotics programming.

Source	Type III Sum of Squares	df	Mean Square	F	Sig	Partial Eta Squared	Decision
Corrected Model	24314.392^a^	4	6078.598	45.731	0.000	0.729	Significant
Intercept	13531.602	1	13531.602	101.802	0.000	0.600	Significant
TPSPreTest_Score	3314.745	1	3314.745	24.938	0.000	0.268	Significant
Method	20690.771	1	20690.771	155.662	0.000	0.696	Significant
Gender	198.456	1	198.456	1.493	0.226	0.021	Not Significant
Method * Gender	214.991	1	214.991	1.617	0.208	0.023	Not Significant
Error	9038.622	68	132.921				
Total	259267.000	73					
Corrected Total	33353.014	72					

a. R Squared = .729 (Adjusted R Squared = .713).

The results in Table 5 reveal that male students taught robotics programming using PARA had a pre-test mean achievement score of (x̅ = 31.60, SD = 12.29) and a post-test mean achievement score of (x̅ = 69.60, SD = 11.50). In comparison, female students had a pre-test mean achievement score of (x̅ = 26.67, SD = 13.55) and a post-test mean achievement score of (x̅ = 52.00, SD = 19.43). Additionally, the adjusted mean scores were 55.80 for male students and 49.80 for female students. These findings suggest that male students taught using PARA achieved slightly higher mean scores than their female counterparts.

**TABLE 5:** Influence of Gender on Achievement Score of Students taught Robotics Programming using PARA.

Gender	N	Pre-test		Post-test		Adjusted mean ( $\left.\bar{x}\right)$
		$\bar{x}$	SD	$\bar{x}$	SD	
Male	20	31.60	12.29	69.60	11.50	55.80
Female	14	26.67	13.55	52.00	19.43	49.80

The results in Table 2 indicate no statistically significant main effect of gender on students’ mean achievement scores in robotics programming, F (1,68) = 6.098, p > 0.05. Consequently, the null hypothesis was not rejected, implying no significant difference in the mean achievement scores of male and female students taught robotics programming using PARA. Furthermore, Table 6 reveals that male students taught using PARA had a pre-test mean task persistence score of (x̅ = 32.30, SD = 7.94) and a post-test mean task persistence score of (x̅ = 70.80, SD = 14.02). In comparison, female students had a pre-test mean task persistence score of (x̅ = 48.33, SD = 14.77) and a post-test mean task persistence score of (x̅ = 78.67, SD = 11.96). Additionally, the adjusted mean scores were 55.92 for male students and 59.67 for female students. These findings suggest that female students taught robotics programming using PARA exhibited a slightly higher mean task persistence score than their male counterparts.

**TABLE 6:** Influence of Gender on Task Persistence Score of Students Taught Robotics Programming using PARA.

Gender	N	Pre-test		Post-test		Adjusted mean ( $\left.\bar{x}\right)$
		$\bar{x}$	SD	$\bar{x}$	SD	
Male	20	32.30	9.45	70.80	14.02	55.92
Female	14	48.33	14.77	78.67	11.96	59.67

The results in Table 4 show no statistically significant main effect of gender on students’ mean task persistence scores in robotics programming, F (1, 68) = 1.493, p > 0.05. Consequently, the null hypothesis was not rejected, suggesting no significant difference in task persistence between male and female students taught using PARA. This implies that both male and female students demonstrated similar levels of task persistence.

Furthermore, the results in Table 7 reveal that male students taught robotics programming using PARA had a higher post-test mean achievement score (x̅ = 69.60, SD = 11.50) than their female counterparts (x̅ = 52.00, SD = 19.43). Meanwhile, male students taught using the IPP method had a post-test mean achievement score of (x̅ = 42.83, SD = 10.16), whereas their female counterparts scored (x̅ = 45.33, SD = 14.88). These findings indicate that male students taught using PARA outperformed their female counterparts in robotics programming. Additionally, the results suggest an interaction effect between instructional method and gender on students’ mean achievement scores in robotics programming.

**TABLE 7:** Interaction effect of teaching methods and Gender on Students’ Mean Achievement Score in Robotics Programming.

Gender	N	PARA		N	IPP	
		$\bar{x}$	SD		$\bar{x}$	SD
Pre-test						
Male	20	31.60	12.29	24	30.50	8.18
Female	14	26.57	13.55	15	31.20	12.31
Post-test						
Male	20	69.60	11.50	24	42.83	10.16
Female	14	52.00	19.43	15	45.33	14.88
Observed $\bar{x}$	34	63.00	16.81	39	43.79	12.68
Adjusted $\bar{x}$		61.78			43.81	

**FIGURE 4:**
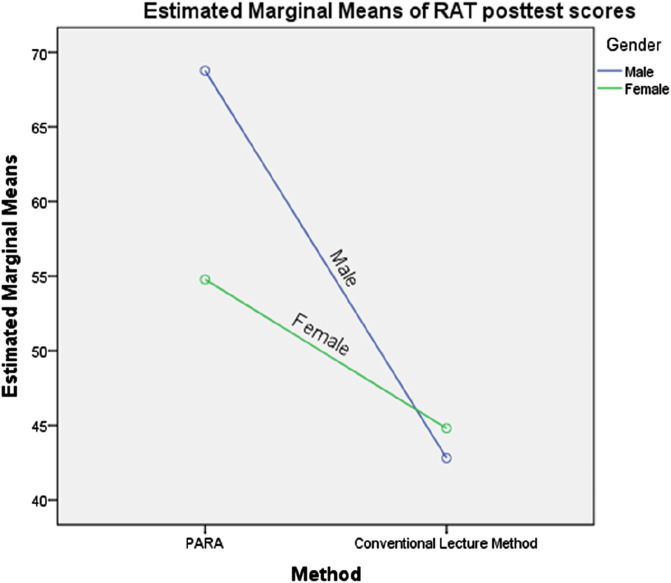
Profile Plot Showing the Interaction of Method and Gender on mean achievement score. Covariates appearing in the model are evaluated at the following values: RAT pretest scores = 30.47.

Table 2 shows a statistically significant interaction effect between instructional method and gender on students’ mean achievement scores in robotics programming, F (1, 68) = 10.519, p < 0.05. The profile plots in Figure 4 illustrate the nature of this interaction, with the intersection of the lines indicating a significant interaction effect. Consequently, the null hypothesis was rejected, confirming that the instructional method and gender jointly influenced students’ achievement in robotics programming.

Table 8 shows that male students taught robotics programming using PARA had a post-test mean task persistence score of (x̅ = 70.80, SD = 14.02), while their female counterparts scored higher with a mean of (x̅ = 78.67, SD = 11.96). In contrast, male students taught using the IPP method had a post-test mean task persistence score of (x̅ = 38.33, SD = 15.18), while their female counterparts had a mean score of (x̅ = 42.67, SD = 10.88). Additionally, a comparison of pre-test and post-test scores for male students taught using the conventional method revealed a decline in task persistence, with a higher pre-test mean score (x̅ = 45.54, SD = 21.56) compared to their post-test mean score (x̅ = 38.33, SD = 15.18). These findings indicate that female students taught robotics programming using PARA demonstrated more remarkable task persistence than their male counterparts. Furthermore, the results suggest an interaction effect between instructional method and gender on students’ mean task persistence scores in robotics programming.

**TABLE 8:** Interaction effect of teaching methods and Gender on Students’ Mean Score on Task Persistence in Robotics Programming.

Gender	N	PARA		Conv. Lect. Method		
		$\bar{x}$	SD	N	$\bar{x}$	SD
Pre-test						
Male	20	32.30	9.45	24	45.54	21.56
Female	14	48.33	14.77	15	39.13	13.88
Post-test						
Male	20	70.80	14.02	24	38.33	15.18
Female	14	78.67	11.96	15	42.67	10.88
Observed $\bar{x}$	34	73.75	13.46	39	40.00	13.70
Adjusted $\bar{x}$		75.40			40.18	

**FIGURE 5:**
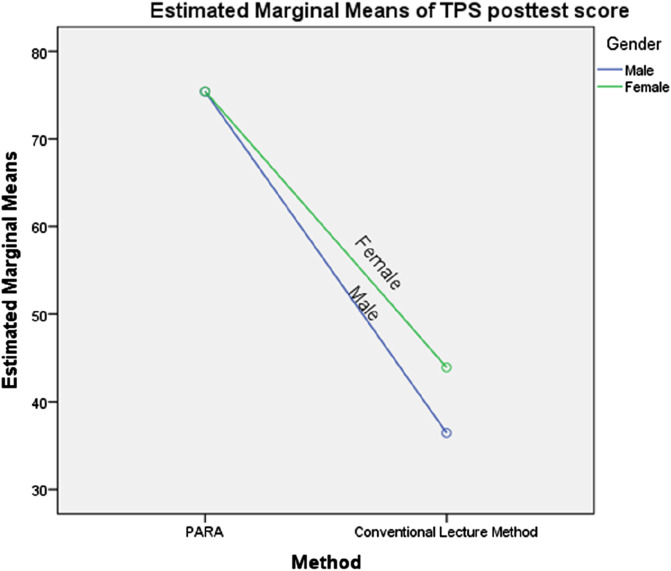
Profile plot showing the interaction of method and gender on mean task persistence score. Covariates appearing in the model are evaluated at the following values: TPS pretest scores = 41.69.

Results in Table 4 show a non-statistically significant interaction effect between teaching methods and gender on students’ mean task persistence ratings in robotics programming, F (1, 68) = 1.617, p > 0.05. The profile plots in Figure 5 illustrate the interaction pattern between method and gender, with the parallel lines on the graph suggesting a lack of significant interaction. Consequently, the null hypothesis was not rejected, confirming that there was no significant interaction effect between teaching methods and gender on students’ task persistence ratings in robotics programming.

## 5 Discussion

### 5.1 Project-based arduino robot application and achievement scores of students in robotics programming

The findings of this study revealed that students taught using the Project-Based Arduino Robot Application (PARA) achieved higher mean scores than those taught using the conventional lecture method. Moreover, the results indicated a significant difference in achievement between students exposed to PARA and those instructed through conventional method (IPP). This outcome can plausibly be attributed to the intervention provided to students in the PARA group. These students had the opportunity to interact with tangible components such as the Arduino Uno microcontroller, light sensor, potentiometer, LCD screen, temperature sensor, PIR motion sensor, and HC-05 Bluetooth module, among others. Engaging with these physical objects likely captured students’ attention, fostering sustained engagement in learning tasks and, consequently, enhancing their achievement.

This finding aligns with Papert’s theory of constructionism, which posits that learning is most effective when individuals actively construct knowledge through interaction with tangible objects, such as robots, in real-world contexts (Papert, 1993; Papert, 1980). Additionally, the PARA platform promotes teamwork through group activities and assignments while also accommodating individualized learning via its assessment component. This dual approach may have further contributed to the significant improvement in students’ achievement compared to those taught through conventional lectures.

The results of this study are consistent with the findings of Marouani (2022), who investigated the application of Arduino in project-based situated engineering teaching. Marouani found that students in the experimental group demonstrated academic improvement, increasing the course mean from 77.1 to 85.3. The ability to actively explore and construct knowledge was attributed to this improvement in academic achievement and interest. Similarly, Papadakis (2020) reported a statistically significant difference in achievement between students who received traditional instruction and those who engaged in a project-based game development approach, with the experimental group outperforming the control group. This further confirms the effectiveness of intervention-based learning in enhancing students’ knowledge of robotics programming.

Likewise, findings from Victal and Candido (2019) support the benefits of hands-on, project-based learning. Their study demonstrated that once all projects functioned as initially intended, students not only developed proficiency in Arduino manipulation but also acquired essential skills in programming languages, assembling, and programming robotic structures. The study highlighted the significant advantages of group-based learning, which were not as prominent in individualized learning environments. Furthermore, Qidwai (2011) found that adopting a project-based approach to robotics courses at the undergraduate level significantly enhanced content dissemination. The structured and focused nature of project-based learning benefited both students and instructors, extending learning beyond curriculum boundaries into real-world applications. This experiential learning approach made the subject more engaging and enjoyable, ultimately improving student achievement.

### 5.2 Project-based arduino robot application and task persistence scores of students in robotics programming

The findings for Research Question 2 show that students taught robotics programming using the Project-Based Arduino Robot Application (PARA) exhibited higher task persistence scores than their counterparts instructed through the conventional lecture method (IPP). Moreover, the results showed a significant difference in task persistence between the two groups, favouring those taught with PARA. This outcome is plausible, as the PARA platform offers students continuous opportunities to interact with robotics kits, allowing them to engage with hands-on tasks at any time. The tasks were intentionally designed to be both educational and enjoyable, enabling students to manipulate real-world objects and control them while executing robotics projects. This experiential learning approach likely stimulated their curiosity and fostered a problem-solving mindset, motivating them to persist in overcoming challenges. The interactive nature of PARA may have further contributed to students’ willingness to engage in extended learning sessions, thereby enhancing their task persistence compared to those taught using traditional methods.

These findings are consistent with the work of Israel-Fishelson and Hershkovitz (2019), who found that intrinsically motivated and mastery-oriented students, those who actively seek to develop their competence and skills while mastering new tasks, exhibit higher persistence, even when faced with challenges. Their study also emphasizes that persistence is influenced by contextual factors such as task difficulty, teacher encouragement, and personal attributes. The PARA platform was specifically designed to make robotics programming more engaging and accessible by reducing the perceived difficulty associated with learning programming. By lowering cognitive barriers and fostering an enjoyable learning experience, PARA likely contributed to the significant increase in task persistence observed among students in this study.

### 5.3 Influence of gender on mean achievement scores of students taught robotics programming using PARA

The findings show that male students taught robotics programming using the Project-Based Arduino Robot Application (PARA) had a slightly higher mean achievement score than their female counterparts. However, the difference was not statistically significant, indicating that gender did not play a major role in students’ achievement when using PARA. This outcome is likely attributable to the instructional strategy that provided equal learning opportunities for all students and actively engaged them in the learning process. The PARA platform facilitated hands-on interaction for both male and female students, offering various engaging and enjoyable activities that captured their attention equally. By ensuring equitable access to learning resources and experiences, the platform may have contributed to the absence of significant gender differences in achievement.

The findings of this study align with previous research that reported no significant gender differences in programming achievement (Zhong et al., 2016; Akinola, 2015; Lau and Yuen, 2009). However, they contradict the findings of Noh and Lee (2020), who reported that female students outperformed their male counterparts in programming achievement. Additionally, the results diverge from those of Ayalew et al. (2018) and Baser (2013), who found that male students demonstrated higher academic achievement in computer programming compared to their female peers.

### 5.4 Influence of gender on mean task persistence scores of students taught robotics programming using PARA

The results showed that female students had a slightly higher task persistence score than their male counterparts when taught robotics programming using the Project-Based Arduino Robot Application (PARA). However, the difference was not statistically significant, indicating that gender did not substantially influence task persistence in this learning context. This suggests that both male and female students demonstrated similar levels of perseverance when engaging with robotics programming through PARA.

The slight advantage observed in female students’ task persistence could plausibly be attributed to social and cultural factors. In many societies, girls are often expected to take on responsibilities such as housekeeping, caregiving, and employment from an early age, which may foster greater persistence when faced with challenging tasks. Parental expectations may also contribute, as girls are often required to remain at home and focus on responsibilities, whereas boys may have more freedom to explore external activities. This conditioning could result in higher perseverance among female students when tackling complex learning tasks. This finding aligns with the study by Torgrimson et al. (2021), which reported that female students were more likely to demonstrate task persistence compared to their male counterparts. However, it contrasts with the findings of Gilligan et al. (2023), who reported that male students exhibited greater persistence on tasks than female students.

### 5.5 Interaction effect of teaching methods and gender on students’ mean achievement scores in robotics programming

The findings from this theme show a significant interaction effect between instructional method and gender on students’ mean achievement scores in robotics programming. Specifically, male students taught using the Project-Based Arduino Robot Application (PARA) outperformed their female counterparts. A plausible explanation for this outcome could be that male students dedicated more time to engaging with activities on the PARA platform. In contrast, female students may have experienced distractions from their male peers. Since the participants were second-year undergraduate students in mid-adolescence, social dynamics may have influenced their focus and engagement with the learning tasks. As a result, male students demonstrated higher achievement than female students. This finding contrasts with the study by Obiajulu (2014), which found no significant interaction effect between instructional method and gender on students’ achievement. It also disagrees with the findings of Noh and Lee (2020), who reported that female students outperformed their male counterparts in academic achievement.

### 5.6 Interaction effect of teaching methods and gender on students’ mean scores on task persistence in robotics programming

The findings of this study revealed no significant interaction effect between teaching methods and gender on students’ mean ratings of task persistence in robotics programming. Although female students taught using the Project-Based Arduino Robot Application (PARA) demonstrated higher task persistence scores than their male counterparts, the difference was not statistically significant. This result aligns with the findings of Torgrimson et al. (2021), who reported no significant interaction effect of method and gender on task persistence, though their study indicated that female students were more likely to exhibit higher persistence than male students. However, the result contradicts Gilligan et al. (2023), who found a significant interaction effect, with male students demonstrating greater persistence on tasks than their female counterparts.

A particularly surprising finding emerged when comparing the pre-test and post-test task persistence scores of male students taught robotics programming using the conventional lecture method. The results showed that their pre-test score (45.54) was higher than their post-test score (38.33), suggesting a decline in task persistence over time. Typically, instructional interventions are expected to improve students’ engagement and motivation; however, in this case, the conventional lecture method appeared to discourage students rather than sustain their interest in learning robotics programming. A possible explanation for this outcome is that programming concepts were presented in a highly abstract and difficult manner, making them challenging to grasp (Karaahmetoğlu and Korkmaz, 2019). Consequently, students may have lost motivation and become less willing to persist in learning robotics programming.

## 6 Conclusion

This study examined the effect of the Project-Based Arduino Robot Application (PARA) on undergraduate students’ achievement and task persistence in robotics programming. The findings established that PARA significantly enhanced both students’ achievement and task persistence in robotics programming. Furthermore, the study revealed gender-related disparities in achievement, depending on the instructional intervention used. Notably, PARA played a crucial role in narrowing the gender gap in achievement, as evidenced by the significant interaction effect between method and gender on students’ mean achievement scores.

The results of this study provide strong empirical support for the constructionist theory, which emphasizes learning through active engagement in problem-solving tasks. Constructionism posits that students develop essential skills by solving real-world problems, making learning more meaningful and effective. The success of PARA in improving students’ achievement and task persistence aligns with this theoretical framework, demonstrating that hands-on, project-based approaches facilitate deeper learning and skill acquisition in robotics programming.

## 7 Educational implications

The findings of this study highlight the importance of adopting effective instructional strategies for teaching robotics programming. Traditional lecture-based methods have proven inadequate in addressing students’ difficulties with programming, often leading to lower persistence and achievement. By contrast, PARA was able to mitigate the perceived difficulty and abstract nature of programming, making it more accessible and engaging for students. This suggests that adopting PARA in robotics programming courses can significantly enhance students’ learning experiences and outcomes, regardless of their initial perceptions of programming difficulty.

For educators, the study underscores the need for pedagogical innovation. Many lecturers continue to rely on conventional lecture methods, such as PowerPoint presentations, which do not adequately address students’ challenges in learning programming. The findings suggest that computer and robotics education lecturers should integrate innovative, student-centred instructional strategies like PARA to enhance engagement and learning outcomes. By shifting from passive instructional methods to interactive, hands-on approaches, educators can better support students in overcoming challenges associated with programming. Additionally, this study highlights the importance of considering gender dynamics in instructional design. While PARA contributed to reducing gender disparities in achievement, the observed differences suggest that further efforts are needed to create an inclusive learning environment that supports both male and female students equally. Future research could explore ways to further optimize instructional strategies to ensure equitable learning experiences.

Finally, it is important to acknowledge that variations in the sample sizes of male and female students in both the treatment and control groups may have influenced the study’s outcomes. Future studies should aim for balanced sample sizes to provide more comprehensive insights into gender-related effects in robotics programming education. This study provides compelling evidence that the use of project-based learning approaches, such as PARA, can significantly improve students’ achievement and task persistence in robotics programming. By adopting innovative instructional strategies, educators can foster a more engaging and effective learning environment, ultimately preparing students with the skills and persistence necessary for success in robotics and programming fields.

## 8 Recommendations

In line with the findings of this study, the researchers made the following recommendations.

1. Educational institutions should consider integrating project-based, hands-on approaches like PARA into robotics programming curricula, as initial evidence suggests it can improve student achievement and task persistence compared to lecture-based methods.
2. Lecturers are encouraged to adopt more student-centred, interactive instructional strategies to enhance engagement and learning outcomes in robotics programming.
3. Robotics programming courses should promote gender-inclusive participation, supported by targeted interventions such as mentorship or group work, to help address observed disparities in task persistence and achievement.

## Data Availability

The raw data supporting the conclusions of this article will be made available by the authors, without undue reservation.
